# Longitudinal study of the immune response and memory following natural bovine respiratory syncytial virus infections in cattle of different age

**DOI:** 10.1371/journal.pone.0274332

**Published:** 2022-09-16

**Authors:** Sara Hägglund, Katarina Näslund, Anna Svensson, Cecilia Lefverman, Hakan Enül, Leonore Pascal, Jari Siltenius, Menno Holzhauer, Alexis Delabouglise, Julia Österberg, Karin Alvåsen, Ulf Olsson, Jean-François Eléouët, Sabine Riffault, Geraldine Taylor, María Jose Rodriguez, Marga Garcia Duran, Jean François Valarcher

**Affiliations:** 1 Department of Clinical Sciences, Swedish University of Agricultural Sciences, Uppsala, Sweden; 2 Ruminant Health Department Royal GD Animal Health, Deventer, The Netherlands; 3 CIRAD, UMR ASTRE, F-34398 Montpellier, France and UMR ASTRE, Univ Montpellier, CIRAD, INRAE, Montpellier, France; 4 Department of Energy and Technology, Swedish University of Agricultural Sciences, Uppsala, Sweden; 5 Université Paris-Saclay, UVSQ, INRAE, VIM, Jouy-en-Josas, France; 6 The Pirbright Institute, Ash Road, Pirbright, Woking, Surrey, United Kingdom; 7 Eurofins-Ingenasa, s.a., C/ Hermanos García Noblejas, Madrid, Spain; Michigan State University, UNITED STATES

## Abstract

Human and bovine respiratory syncytial virus (HRSV and BRSV) are closely genetically related and cause respiratory disease in their respective host. Whereas HRSV vaccines are still under development, a multitude of BRSV vaccines are used to reduce clinical signs. To enable the design of vaccination protocols to entirely stop virus circulation, we aimed to investigate the duration, character and efficacy of the immune responses induced by natural infections. The systemic humoral immunity was monitored every two months during two years in 33 dairy cattle in different age cohorts following a natural BRSV outbreak, and again in selected individuals before and after a second outbreak, four years later. Local humoral and systemic cellular responses were also monitored, although less extensively. Based on clinical observations and economic losses linked to decreased milk production, the outbreaks were classified as moderate. Following the first outbreak, most but not all animals developed neutralising antibody responses, BRSV-specific IgG1, IgG2 and HRSV F- and HRSV N-reactive responses that lasted at least two years, and in some cases at least four years. In contrast, no systemic T cell responses were detected and only weak IgA responses were detected in some animals. Seronegative sentinels remained negative, inferring that no new infections occurred between the outbreaks. During the second outbreak, reinfections with clinical signs and virus shedding occurred, but the signs were milder, and the virus shedding was significantly lower than in naïve animals. Whereas the primary infection induced similar antibody titres against the prefusion and the post fusion form of the BRSV F protein, memory responses were significantly stronger against prefusion F. In conclusion, even if natural infections induce a long-lasting immunity, it would probably be necessary to boost memory responses between outbreaks, to stop the circulation of the virus and limit the potential role of previously infected adult cattle in the chain of BRSV transmission.

## Introduction

Human and bovine respiratory syncytial virus (HRSV and BRSV) causes respiratory infection in their respective host and have similar virus characteristics and pathogenesis. Understanding the duration and character of protective immunity to these viruses is essential to develop effective vaccines and to tailor vaccination schemes to susceptible individuals, in order to stop virus circulation.

Infections with HRSV in humans are very common worldwide. About 60–70% of all children become infected during their first year of life [1] and nearly one third of all hospitalised children under one year of age are hospitalised due to HRSV [2]. Reinfections are very frequent. Although the clinical symptoms are milder upon each reinfection, virus shedding occurs, albeit in lower quantities and with less duration [3]. This partial protection could partly be explained by the existence of two antigenic subgroups (HRSV A and B) and multiple genotypes [4], but repeated reinfections with homologous virus is also possible, indicating that immune responses are often quite weak [5].

Both the humoral and the cellular responses are crucial in preventing infection and eliminating HRSV, however, as a single parameter, virus-neutralising antibody titres in serum are those most commonly (negatively) correlated both to severity of disease and occurrence of reinfection [6–8]. Indeed, children develop weak virus-neutralising antibody responses and can be susceptible to HRSV within a year after a primary infection [9, 10]. Some children develop a detectable T lymphocyte memory, but this does not appear to be sufficient to afford a long-term protection [11]. Overall, since HRSV is ubiquitous, and since repeated reinfections are inevitable and not always detectable, long term studies on the kinetics of antibody responses to a controlled number of natural HRSV infections are difficult to perform.

The situation in the cattle population is somewhat different. Cattle do not mix to the same extent as humans and some herds have little exchange of pathogens with other herds, at least in Scandinavia. In addition, the genome of the bovine virus is more conserved than its human counterpart and the antigenic differences are smaller [12, 13]. Despite this limited degree of variation, BRSV is a major viral pathogen and of great concern. The virus is diagnosed in 12% to 83% of respiratory disease outbreaks in European cattle [14, 15]. In areas and herds with frequent BRSV circulation, calves are more prone to disease than adults, indicating that a protective or a partially protective immunity is acquired [16]. This agrees with data from experimental infections, which suggest that both clinical and virological protection to homologus virus last at least four months [17]. However, little is known about the field situation, if and when cattle can be naturally re-infected and consequently shed virus, with no or mild clinical signs. This information would be essential to understand if previously infected animals would need to be vaccinated to rupture the viral cycle of transmission, and at which point after a primary infection this would be required.

The main objective of this study was to characterise the long-term kinetics of immune responses against natural BRSV infections in animals of different age. To enable consideration of these results in other contexts and herds in the future, we additionally wanted to describe the clinical pattern and the impact of the BRSV infections on milk production in the studied cattle population. Furthermore, to investigate the memory and protective immunity to BRSV, virus shedding and humoral responses were monitored in cows that were repeatedly exposed to BRSV, four years apart.

## Materials and methods

### Description of the herd

In January 2016, when a BRSV outbreak occurred, Lövsta research farm at the Swedish University of Agricultural Science consisted of 534 cattle, mainly of the Holstein breed (41%) and the Swedish red and white breed (57%). The average age was 33 months, and most cows were in parity 1 (34%), 2 (26%) and 3 (21%). The lactating dairy cows (n = 204) were housed in free-stalls, in a heated cubicle system with approximately 50–60 animals per group, in the same section of a building. They were milked in automatic milking systems, either by voluntary robot milking (DeLaval VMS^™^, average 2.3 times/ day), or twice a day in a milking rotary parlour (DeLaval AMR^™^). The average yearly milk production per cow was 9971 kg energy corrected milk (ECM). Dry cows and heifers older than 5.5 months were also housed in free-stall cubicles, in groups of 20–25 individuals, in a separate section of the building. From two weeks before calving, dry cows were moved to a separate compartment for calving. Cattle that needed close surveillance and special care were housed in a sick ward. Weaned calves were housed in group pens on straw in four different rooms and calves up to eight weeks of age were housed outdoor, singly or in pairs in covered calf hutches under a roof. None of the animals were vaccinated against BRSV, before 2016 or during the entire study.

At the time of a second BRSV outbreak in March 2020, the same herd consisted of 560 cattle, 471 (83%) of which were born after the first outbreak. The number of lactating cows and the average milk production had increased to 285 and 10313 kg ECM per year, respectively, and the average replacement rate had increased from 35% to 43%. The farm was self-sustainable regarding replacement of cows and had not introduced any cattle from other farms since 2011. Sweden is free from bovine herpes virus type 1, bovine leucosis virus and bovine viral diarrhoea virus (since 1995, 2001 and 2014, respectively).

### Data collection regarding food intake and milk production of cows that continued to produce milk for commercial purpose

Data on sold milk and the composition of this milk was collected from reports of the dairy company (Arla Foods) in both 2016 and 2020. In addition, the daily milk production and daily roughage and non-roughage intake of 272, 163 and 272 cows, respectively, was collected for December 1, 2015 to January 31, 2016. These animals were fed roughage in weighted feed troughs and non-roughage in automatic feeders and the milk was weighed in the milking systems.

Cows with the most severe clinical signs were placed in the sick ward, where neither milk nor food intake was recorded. Data from cows at these time points were thus missing and, consequently, the estimation of food intake and milk production during the outbreak was valid only for cows without severe clinical signs of disease. Considering that most clinical disease occurred during a period of two weeks, the study period was subdivided into sub-periods (phases) according to Table 1.

**Table 1:** Time periods used to study changes in food intake and milk production during a BRSV outbreak.

Start date	Stop date	Phase
December 1, 2015	December 14, 2015	Pre
December 15, 2015	December 22, 2015	Base
December 23, 2015	January 2, 2016	Inter
January 3, 2016	January 15, 2016	Outbreak
January 16, 2016	January 31, 2016	Post

### Sample collection

In January 2016, during the first outbreak, nasal secretions and blood samples were collected from 10 animals with clinical signs (Table 2). Between March 2016 and February 2018, samples were additionally repeatedly collected from 33 female cattle of the Swedish red and white breed, in different cohorts based on age (hereafter called closely monitored individuals, see Table 2). Besides age, these animals were selected based on having a good health record, as well as on gender and genetic background, to increase the probability that they would be present in the herd for the entire study period. Blood and milk (if applicable) were collected every second month. Nasal secretions were similarly collected from 5 of these individuals, aged 4–5 months at the time of the outbreak.

**Table 2:** Sampling for BRSV diagnosis and monitoring of BRSV-specific immunity.

Sample occasion	Age at outbreak 2016	Sample type	Number of individuals sampled (cattle id^a^)
**Outbreak 2016**	2–9 m	Serum NS	7 (560, **609, 618, 620, 627, 629, 636**)
	31–60 m	Serum NS	3 (155, 287, 1664)
**Repeated sampling 2016–2018 (closely monitored animals)**	Born during or within 3 w after the outbreak 2016	Serum	5 (**662, 666, 672, 675, 676**)
	2–3 m	Serum	6 (**620, 624, 627, 629, 634, 636**)
	4–5 m	Serum NS	6 (6**03, 604, 605, 606, 609, 618**)
	7–11 m	Serum Milk	8 (**546, 548, 552, 554, 555, 580, 581, 583**)
	23–30 m (early gestation, < 6 m)	Serum Milk	4 (**306, 355, 403, 415**)
	23–30 m (late gestation, > 6.5 m)	Serum Milk	4 (**372, 373, 377, 399**)
**26–27 m after outbreak 2016**	Born at least 5 m. after the outbreak 2016	Serum	50 (random, aged 3 d to 22 m at sampling)
**3 w before outbreak 2020**	See above	Serum	9 (**355, 580, 583, 606, 636, 666, 672, 675, 676**)
**Outbreak 2020**	Born > 2 m before outbreak 2016	NS	7 (**355**, 436, 553, **580, 605, 606, 636**)
	Born > 9 m after outbreak 2016	NS	5 (777, 778, 784, 842, 2173)
**2.5 m after outbreak 2020**	See above	Serum	8 (**580, 583, 606, 636, 666, 672, 675, 676**)

^a^ Cattle id in bold if animal was closely monitored (repeatedly sampled)

NS, nasal swab; m, month(s); w, week(s); d, day(s)

To reinforce the monitoring of absence of new BRSV infections, blood was additionally collected 26–27 months after the outbreak, from 50 other animals that were born after the outbreak in 2016. In February 2020, during a new upsurge of BRSV outbreaks in the geographical area where the university farm was situated, the animal care takers within the farm collected blood from closely monitored individuals that were still alive and accessible. Three weeks later, in March 2020, the second BRSV outbreak occurred. Nasal swabs were then collected from five of the closely monitored animals, two additional cows that had been present during the outbreak in 2016 and from five younger cows (born after September 2016). Blood was additionally collected from eight of the closely monitored animals 2.5 months later (Table 2). The study was approved by the Ethical Committee of the district court of Uppsala, Sweden (Ref. no. C146/15 and 5.8.18-16188/2017). The herd is an approved research facility (dnr 5.2.18-870707/4) and a contract including a written consent was made before the start of the study.

### Sampling, sample preparation and storage

Blood was collected by using BD vacutainer^®^ systems with clot activator tubes and heparinized tubes. Nasal secretions were either obtained by nasal swabs (virus swab UTM^™^, Copan, Italy) that were frozen at −75°C until RNA extraction for virus detection, or was collected by using tampons left for 5–10 min in one nostril. The tampons were centrifuged at 200 x *g* and room temperature (RT) for 10 min and the secretions were frozen at −20°C until antibody analysis. Peripheral blood mononuclear cells (PBMC) were isolated from heparinized blood as described previously [18] and were immediately frozen at −80°C in foetal calf sera (FCS) containing 10% dimethyl sulfoxide. Serum was extracted from blood by centrifugation at 3000 x *g* at RT for 10 min and was frozen at −20°C. Milk was defatted by centrifugation at 3000 x *g* and RT for 10 min and was frozen at −20°C.

### Virus detection

Analyses for BRSV-RNA were performed on nasal swab medium by Taqman RT-qPCR using LSI VetMax^™^ Screening Pack–Ruminants Respiratory Pathogens (Life technologies, France), after extraction by using the RNAeasy^®^ Mini kit (Qiagen, Sweden) according to the manufacturers’ instructions. A standard curve based on a BRSV-infected cell lysate with a known titre was used for virus quantification. Live BRSV was isolated on bovine turbinate cells, as previously described [19].

### BRSV-specific immune responses

Analyses for BRSV-specific IgG, IgG1, IgG2 and IgA antibodies were performed by indirect (IgG, IgG1, IgG2) and capture (IgA) ELISAs, as described previously [19]. Antibodies reactive against the F protein were analysed by a competitive ELISA based on HRSV and mAb 56F, reactive against the antigenic site IV (Ingezim Compac, INGENASA), according to the manufacturers’ instructions, and N-specific IgG1 antibodies were analysed by indirect ELISA, as described previously [20], but with slight modifications. In brief, 96-well plates were coated overnight with 100 ng per well of purified HRSV N protein or an irrelevant purified protein (control antigen), diluted in carbonate buffer (pH 9.6), at 4°C. After blocking with 3% bovine serum albumin (BSA) in PBS for 1 hour at RT, the sera were added at 1/25 dilutions and incubated for 1h at 37°C. The bound antibodies were detected using horseradish peroxidase (HRP)-labelled monoclonal anti-bovine IgG1 (EC10, INGENASA) incubated for 30 minutes at 37°C. Washes between consecutive steps were performed with 0.05% Tween 20 in PBS. The substrate and stopping solution consisted of 3,3’,5,5’-tetramethylbenzidine (TMB)-MAX (Neogen Corporation, Lexington, KY) and 0.5 M sulfuric acid. Absorbance was measured at 450 nm in a Multiscan Ascent ELISA reader. For each serum sample, the optical density (OD) against the control antigen was subtracted from the OD value against the N protein (Corrected OD, COD) and the COD was transformed into sample-to-positive values (SPs) by using the formula SP = COD_sample_/ (CODpos—COD_neg_).

Analyses for BRSV pre-fusion (PreF)- and post-fusion (PostF)-specific IgG1 antibodies were performed by indirect ELISAs. Briefly, Ni-NTA HisSorb plates (Qiagen, Hillerod, Denmark) were coated overnight with 2 μg/ml his-tagged antigen (kindly provided by P. Kwong, NIAID, NIH, Washington, USA) in PBS containing 0.2% BSA and were thereafter blocked with Sea Block buffer (Thermo Fisher, Gothenburg, Sweden) for one hour at RT. Serial dilutions of sera were added and were visualized by HRP-conjugated monoclonal anti-bovine IgG1 antibodies and TMB-substrate (both from Svanovir, Svanova Indical, Uppsala, Sweden). Antibody-incubations were performed for one hour at RT and washes between consecutive steps were performed with 0.05% Tween 20 in PBS.

Antibodies specific for BRSV PreF were additionally analysed by a competitive ELISA. Briefly, plates were coated with recombinant PreF protein (kindly provided by P. Kwong, NIAID, NIH, Washington, USA) and blocked as above, but after the incubation of sera and a washing step, humanized monoclonal antibodies to PreF (AM14, kindly provided by P. Kwong, NIAID, NIH, Washington, USA) was added. The AM14-antibodies were visualised with HRP-conjugated mouse anti-human IgG1 antibodies (Invitrogen A-10648, clone HP6069) and TMB-substrate (Svanovir, Svanova Indical, Uppsala, Sweden). Antibody incubation and washing steps were performed as above.

BRSV-neutralising antibodies were detected by using Vero cells and BRSV (Strain ATue51908), expressing green fluorescent protein. Briefly, Vero cells were seeded in 96-well plates, at 15 000 cells per well, in cell culture medium (DMEM, Lonza, Belgium) containing 10% FCS. On the following day, serum samples were serially diluted in DMEM without FCS, were mixed with 300 plaque forming units of green fluorescent protein -BRSV and incubated at RT for one hour. The Vero cells were rinsed once with DMEM without FCS and were thereafter inoculated with the serum-virus samples at 37°C and 5% CO_2_ for one hour. Thereafter, DMEM with FCS was added to reach a final FCS concentration of 2%, the cells were incubated at 37°C and 5% CO_2_ for 3 days, and were examined by using a fluorescent microscope (Nikon Eclipse Ts2R). Each serum was tested in eight dilutions, and the titre (defined as the serum dilution needed to completely inhibit 300 plaque forming units) was calculated by linear regression.

BRSV-specific T cells in PBMC were analysed using a lymphoproliferation assay, as previously described [21], but using both live and heat-inactivated BRSV (strain DK9402022, kindly provided by prof LE Larsen, Denmark) as antigen for *in vitro* restimulation of the cells. Furthermore, BRSV-specific IFNγ-producing T cells in PBMC were analysed by ELISpot as previously described [22].

### Statistical methods

The mean value for milk production, roughage consumption and non-roughage consumption was calculated for each cow, for each period. These mean values were used in mixed models, which take missing values into account [23]. The models included sub-period as a fixed factor and used cow, and the cow*period interaction, as random factors. Denominator degrees of freedom were estimated according to Kenward and Roger (1997) [24]. The assumptions underlying the models were checked using diagnostic plots. No apparent deviations from normality or homoscedasticity could be detected. Post-hoc pairwise comparisons were adjusted for multiplicity using Tukey’s method and adjusted p values <0.001 was considered statistically significant. The Mixed procedure of the SAS (2018) package was used. Data concerning antibody responses and virus shedding were analysed by using Minitab 18 and two-sided one-way ANOVA followed by Tukeys test, or two-tailed Student T-test.

## Results

### BRSV diagnosis and virus shedding

Bovine respiratory syncytial virus was diagnosed by serology and virus detection in both respiratory outbreaks in the herd. In 2016, the virus was detected in nasal swabs from 9/10 sampled cattle, by RT-qPCR and virus isolation. In 2020, BRSV was detected by RT-qPCR in nasal swabs from 12/12 sampled cows (all sampled once, on the same day and in the same section of the loose range system), including in seven cows that had been present in the herd during the outbreak in 2016. However, the samples from these seven cows contained lower virus quantities than samples from five younger and previously naïve cows (0.41–2.04 (mean 1.33) log 10 TCID_50_ equivalents and 2.31–4.96 (mean 3.79) log 10 TCID_50_ equivalents, respectively, p<0.001, S1 Fig). In 2020, the virus was additionally detected by RT-qPCR in environmental swabs from the food tray of a milk robot but not in a watering cup (data not shown).

### Respiratory outbreak pattern and clinical signs

The first outbreak started on the 3^rd^ of January 2016, when a second parity cow (10 days in milk) had elevated rectal temperature and showed clinical signs of respiratory disease. Within a week, respiratory disease was observed in all stable sections in the building and two weeks later, the calves in the outdoor hutches became affected.

The clinical signs varied. Several animals developed fever (up to 41.1°C), inappetence and a decrease of milk production. A large proportion of the animals had serous to mucopurulent nasal discharge. Some additionally demonstrated coughing, tachypnea and dyspnea, and a few animals had severe dyspnea, anorexia and apathy. The highest morbidity and the most severe clinical signs were observed in lactating cows, *in peripartum* animals in the calving room and in two to six months old calves. The seemingly least affected animals were the youngest calves in the outdoor hutches. Only a few of these were affected, with fever and mild respiratory signs. None of the calves were treated and none died, despite the outdoor temperature being very low (down to −25°C and −15°C during night and day, respectively). Twenty-six adult cattle were treated with non-steroid anti-inflammatory drugs and six of these additionally with procaine benzyl penicillin. One was a pregnant heifer (aged 26 months) and 25 were cows, in parity 1 (n = 12), 2, (n = 2), 3 (n = 9), 4 (n = 1) and 5 (n = 1), which had calved 2–327 (mean 87) days earlier. One newly dried 3-year old otherwise healthy cow, and one 5-year old cow in otherwise poor health died. Overall, clinical signs were observed in the herd for approximately 3 weeks.

The second outbreak started in a similar manner, with disease in adult cows, but with clinical signs first observed on the 11^th^ of March 2020. Overall, this outbreak was slightly milder than that in 2016, but several animals, both young cows and heifers, were moderately to severely affected. All the five closely monitored cows that were accessible for collection of nasal secretions in 2020 had mild clinical signs of respiratory disease, such as nasal discharge and cough. The five younger and previously naïve cows that were also sampled for virus detection in 2020 (that were born after the first outbreak) had mild to severe clinical signs, including tachypnea, dyspnea and pyrexia.

### Impact of BRSV on food intake and milk production

**Fig 1:**
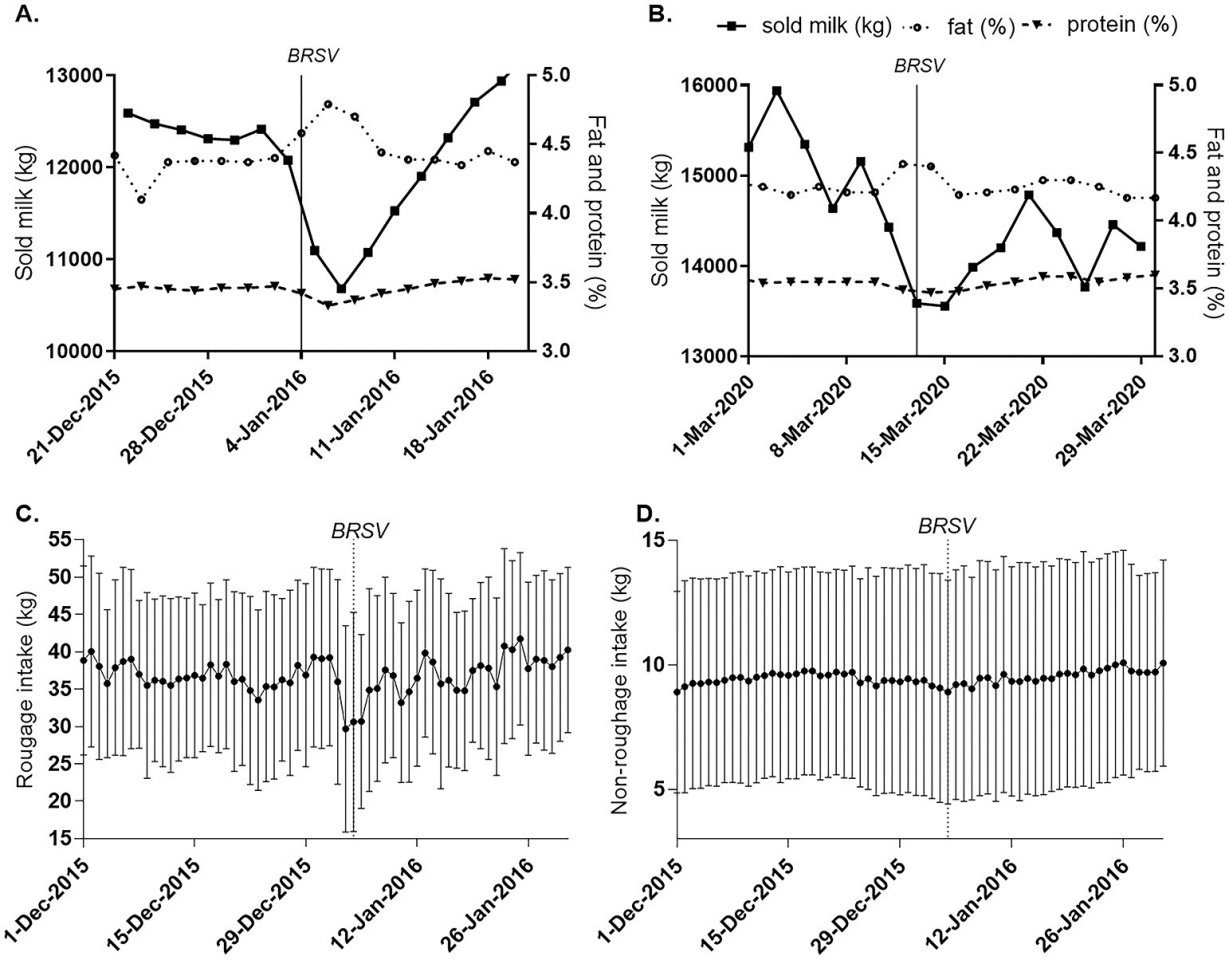
Sold milk, milk composition and feed consumption of cows during BRSV outbreaks in a dairy herd. Weight of sold milk and milk fat and protein composition in (A) 2016 and (B) 2020. Mean (±SD) (C) roughage and (D) non-roughage consumption of cows while remaining in production 2016 (i.e. not moved to sick ward). Data on sold milk are expressed as means of two adjacent days.

During both outbreaks, the fat content increased in sold milk and the volume of sold milk decreased (Fig 1A and 1B). In 2016, when individual food and milk recordings were performed, cows that were healthy enough to remain in the production system had lower milk production and food intake during the outbreak, compared to before and after the outbreak (S1 Table). Given that only data with p-values <0.01 were considered significant; milk production was significantly lower during the outbreak, compared to all other sub-periods, before and after the outbreak, and was lower after compared to before the outbreak (S2 Table). Accordingly, the roughage and non-roughage consumption was significantly lower at the outbreak compared to at least two other subperiods (Fig 1C and 1D, S2 Table).

### Immune responses to BRSV

#### BRSV-neutralising antibody in serum

**Fig 2:**
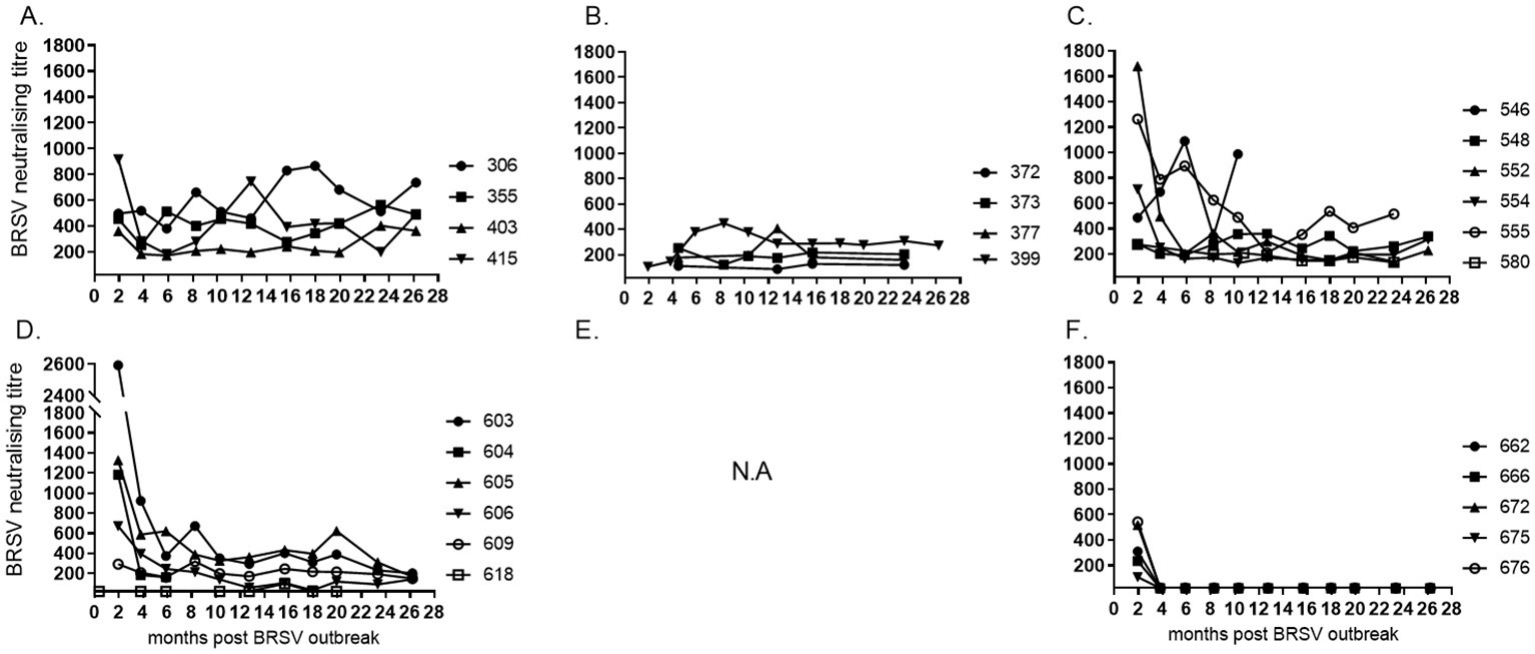
Kinetics of BRSV-neutralising serum antibody titres in cattle of different age and production status. Cattle were (A) 23–30 months old (<6 months gestation), (B) 24–26 months old (>6.5 months gestation), (C) 7–11 months old, (D) 4–5 months old, (E) 2–3 months old, not analysed (N.A), or (F) born during or just after a BRSV outbreak in January 2016 (month 0). Limit of detection in the virus neturalising assay: titre 20.

BRSV-neutralising antibodies were detected 2 months after the outbreak in serum of most animals (Fig 2). Furthermore, from 4 months after the outbreak, the titres of these antibodies remained mostly very stable throughout two years (Fig 2A–2D). In the youngest calves, maternally derived BRSV-neutralising antibodies were detectable at 2–7 weeks of age, but not at 3–4 months of age, nor at any other time point throughout the two first years of life (4–26 months after the outbreak, Fig 2F).

The strongest BRSV-neutralising responses were detected in some of the heifers that were 5–11 months old at the outbreak, but these titres dropped within 4–6 months (animals no. 552, 555, 603, 604 and 605, Fig 2C and 2D). In contrast, one calf that was four months old at the time of the outbreak developed a very poor neutralising antibody response, which was only barely detectable on one occasion, despite that the assays were repeated (calf no. 618, Fig 2D). This calf had nevertheless been infected, since BRSV had been isolated from its nasal secretions and a seroconversion was detected by ELISA, as described below.

When excluding presumably non-infected calves with maternally derived antibodies (MDA) from calculations, no significant differences in mean titres were detected between different age groups, apart from the oldest cows that were in early pregnancy (Fig 2A) which had significantly higher titres than younger cows (Fig 2B and 2C) at 26 months post outbreak (p<0.05).

### BRSV-specific IgG1 antibody in serum

**Fig 3:**
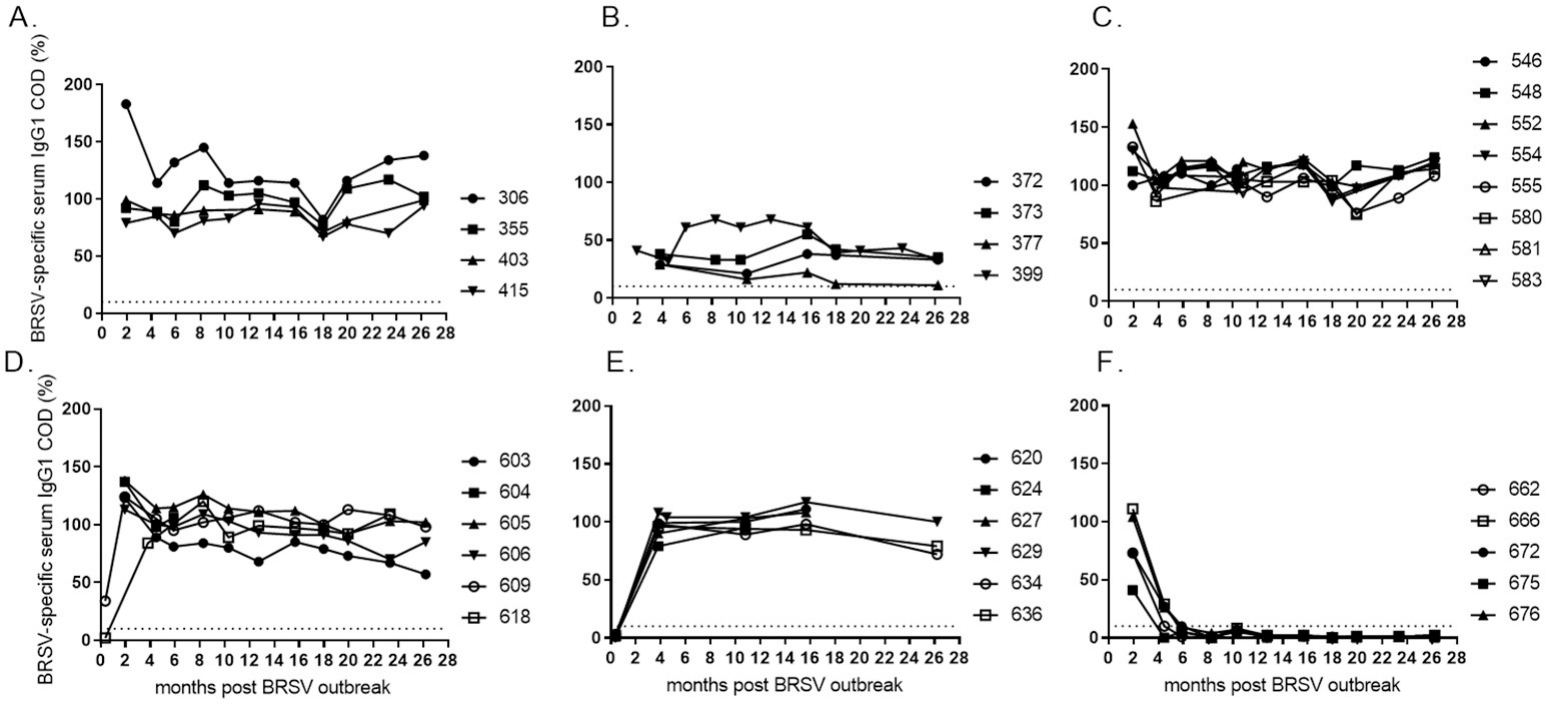
Kinetics of BRSV-specific serum IgG1 in cattle of different age and production status. At the time of a BRSV outbreak the cattle were (A) 23–30 months old (<6 months gestation), (B) 24–26 months old (>6.5 months gestation), (C) 7–11 months old, (D) 4–5 months old, (E) 2–3 months old, or (F) born during or just after the outbreak in January 2016 (month 0). Corrected optical density (COD) values are presented as percentage of a positive control serum.

All cattle that were born before the outbreak in 2016 (including calf 618) responded with BRSV-specific serum IgG1, which remained stable throughout two years (Fig 3A–3E). Four cows that were infected near calving (gestation month >6.5) had significantly weaker responses than four cows infected during earlier pregnancy (gestation month <6), at all three time points when these eight cows were sampled simultaneously (month 15 p<0.01, month 17 p<0.01 and month 25 p<0.001, Fig 3A and 3B).

The youngest cattle, which were born during or just after the outbreak in 2016, became negative for BRSV-specific serum IgG1 within 4–6 months after the outbreak (within 3–5 months of age). Thereafter, they remained seronegative, inferring that they had MDA and that no reinfections occurred (Fig 3F).

### BRSV-specific IgG2 antibody in serum

**Fig 4:**
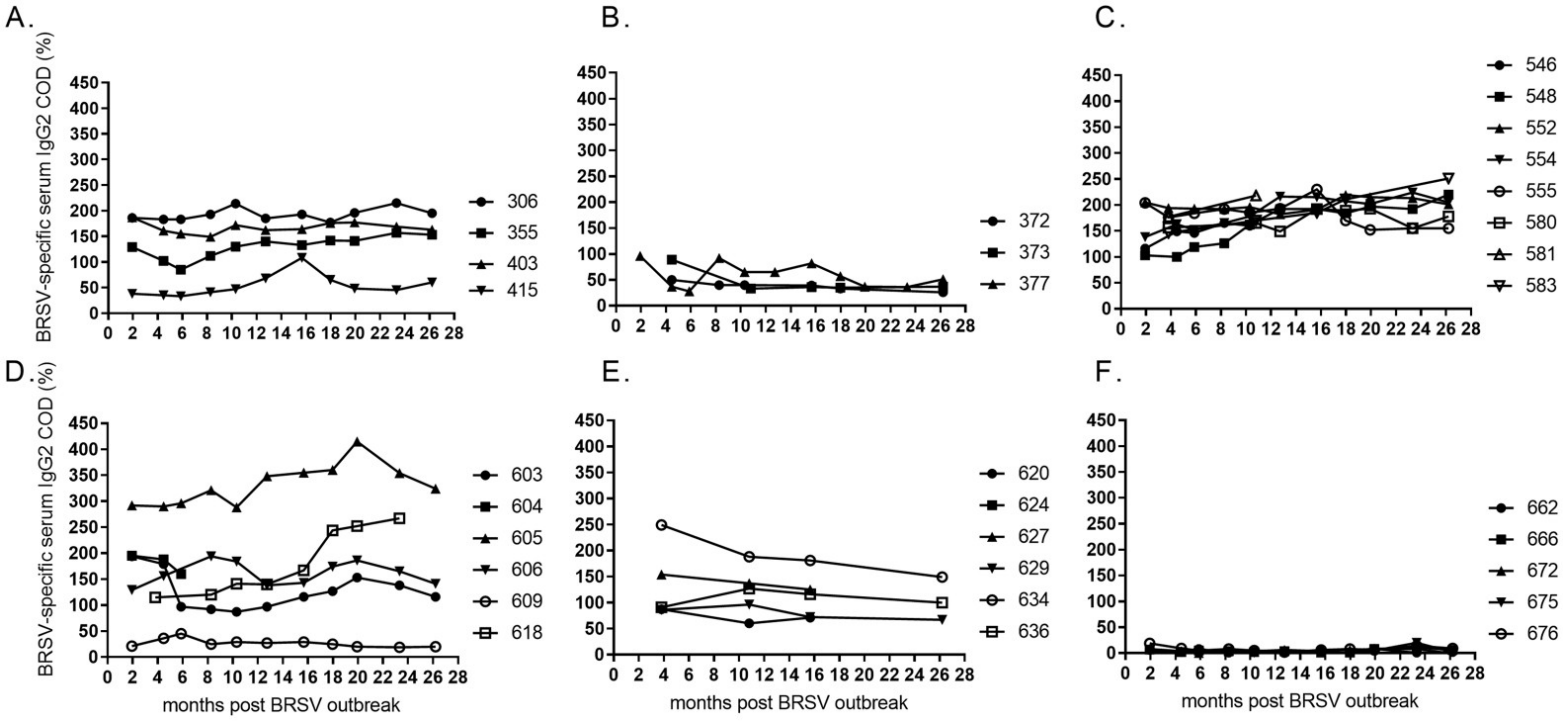
Kinetics of BRSV-specific serum IgG2 in cattle of different age and production status. At the time of a BRSV outbreak the cattle were (A) 23–30 months old (<6 months gestation), (B) 24–26 months old (>6.5 months gestation), (C) 7–11 months old, (D) 4–5 months old, (E) 2–3 months old, or (F) born during or just after the outbreak in January 2016 (month 0). Corrected optical density (COD) values are presented as percentage of a positive control serum.

In contrast to the BRSV-neutralising antibody titres that decreased rapidly in the animals with the strongest responses and thereafter remained at similar levels in most animals, BRSV-specific IgG2 levels did not show a decrease overall, but varied between individuals (Fig 4). Calf no. 618, which developed poor neutralising antibodies, responded well with BRSV-specific IgG2, whereas calf no. 609, which developed moderate titres of neutralising antibodies and high levels of BRSV-specific total IgG1 responded only poorly with BRSV-specific IgG2 (Figs 2D, 3D and 4D). This animal had been infected, since BRSV had been isolated from its nasal secretions.

The strongest BRSV-specific IgG2 response was detected in calf no. 605 (Fig 4D). Similarly, calf 634 was the best IgG2 responder within her age group (Fig 4E). In summary, these data illustrate that IgG2 responses can vary a lot within the same outbreak, even in infected animals of similar age. The highest variability was observed in 2–5 months old calves, and in cows that were not in late gestation (Fig 4A, 4D and 4E).

### HRSV F-reactive antibody in serum

**Fig 5:**
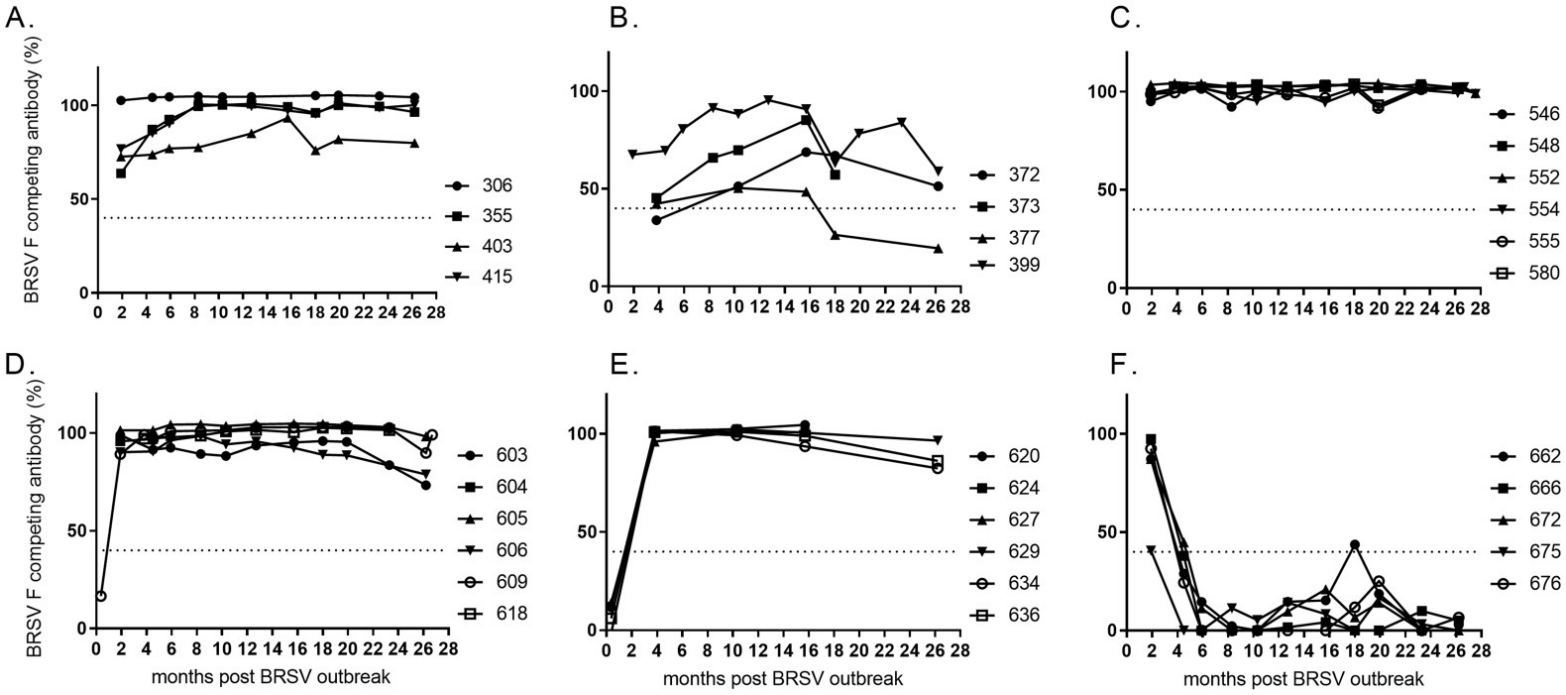
Kinetics of HRSV-F-reactive serum antibodies in cattle of different age and production status. At the time of a BRSV outbreak the cattle were (A) 23–30 months old (<6 months gestation), (B) 24–26 months old (>6.5 months gestation), (C) 7–11 months old, (D) 4–5 months old, (E) 2–3 months old, or (F) born during or just after the outbreak in January 2016 (month 0). Data are presented as competition percentage. The limit of detection is presented as a dotted line.

Since antibodies specific to the F-protein may be neutralising, the dynamics of F-reactive antibodies was investigated by competitive ELISA. The monoclonal antibodies with which serum antibodies competed in this assay recognise the antigenic site IV on the HRSV F protein. Data between both virus-neutralising and F-competing assays agreed to some extent. Indeed, cow 403 had the lowest level of both neutralising and F-competing serum antibodies within its group (Figs 2A and 5A) and cow 399 had the highest level of both neutralising and F-competing serum antibodies within its group (Figs 2B and 5B). On the other hand, cow 618, which barely had any detectable neutralizing antibody response, had high levels of F-competing antibodies throughout the 2 years after infection (Figs 2D and 5D).

### HRSV N-specific IgG1 antibody in serum

**Fig 6:**
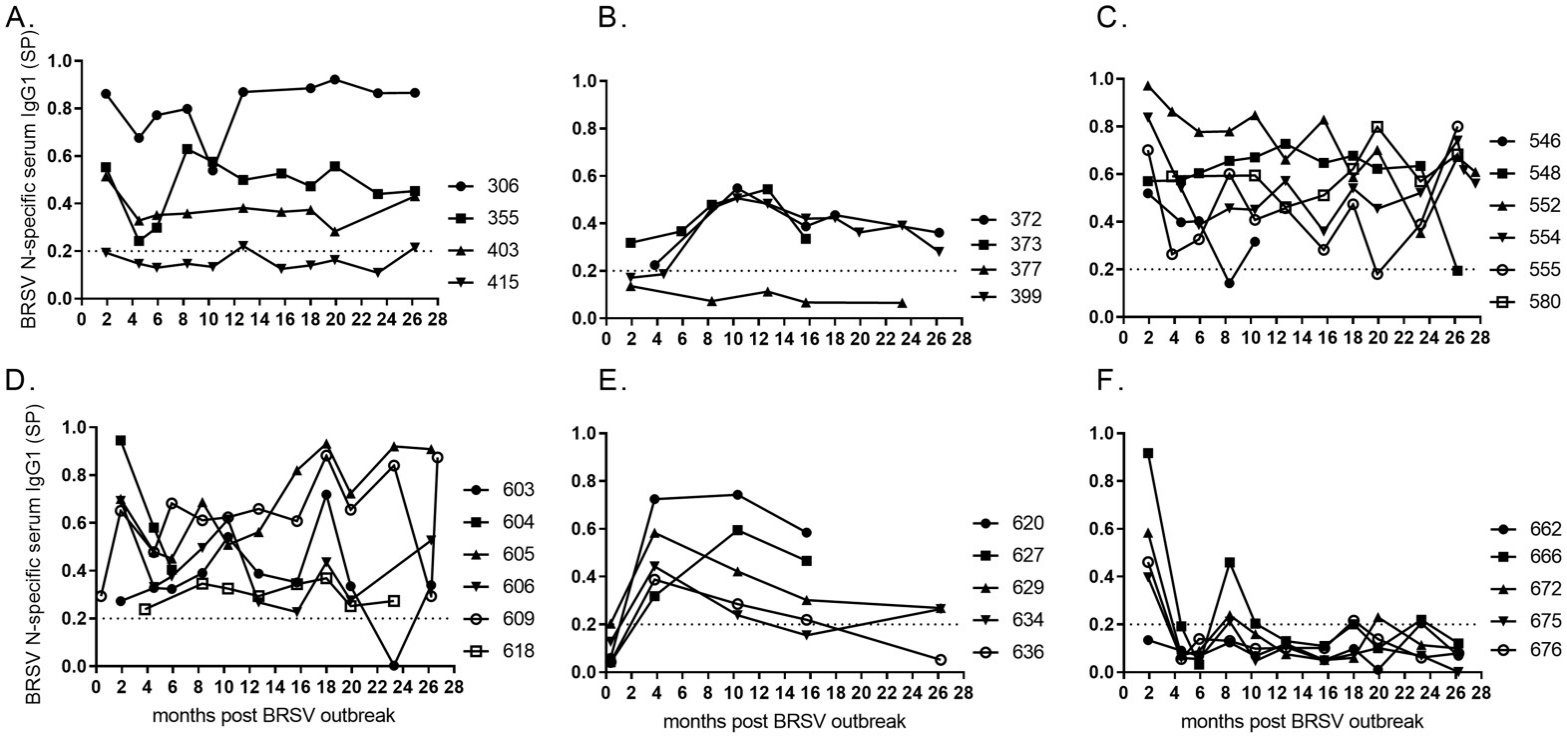
Kinetics of HRSV N-specific serum IgG1 antibodies in cattle of different age and production status. At the time of a BRSV outbreak the cattle were (A) 23–30 months old (<6 months gestation), (B) 24–26 months old (>6.5 months gestation), (C) 7–11 months old, (D) 4–5 months old, (E) 2–3 months old, or (F) born during or just after the outbreak in January 2016 (month 0). For each serum sample, the optical density (OD) against a control antigen was subtracted from the OD value against the N protein (Corrected OD, COD) and the COD was transformed into a sample-to-positive value (SP) by using the formula SP = COD_sample_/(COD_pos_—COD_neg_).

Together with the development of subunit BRSV DIVA vaccines, a DIVA protein needs to be identified. Since the N protein was previously shown to be immunogenic and highly conserved [12, 25] between bovine and human RSV, the dynamics of antibody responses to the HRSV-N protein were investigated. Except for two adult cows (id 415 and 377, Fig 6A and 6B), all animals above 1.5 months of age developed long lasting HRSV N-specific serum IgG1 antibodies. Only a few unspecific, cross-reactive or polyreactive responses were identified, such as in calf no 666 (Fig 6F).

### BRSV-specific IgA antibody in serum

**Fig 7:**
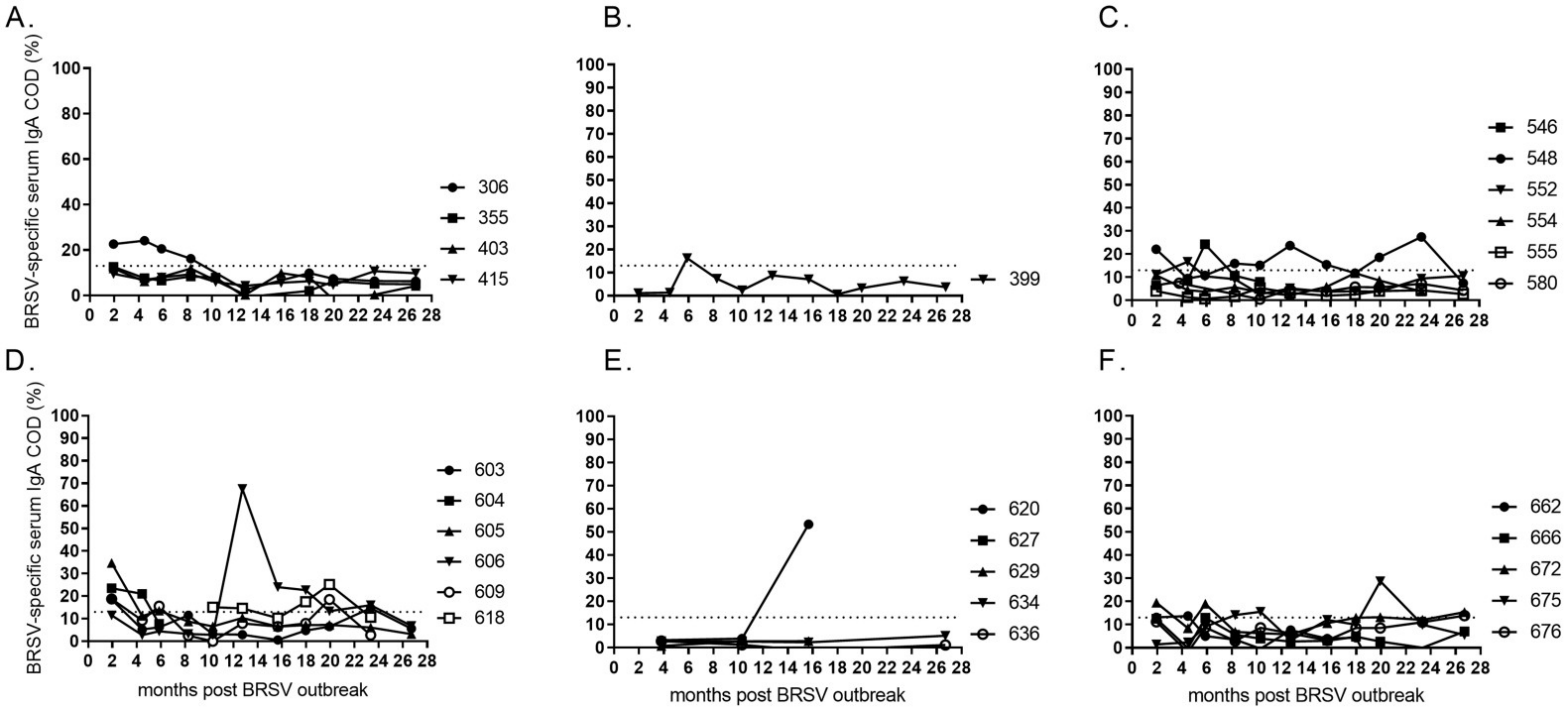
Kinetics of BRSV-specific serum IgA in cattle of different age and production status. At the time of a BRSV outbreak the cattle were (A) 23–30 months old (<6 months gestation), (B) 24–26 months old (>6.5 months gestation), (C) 7–11 months old, (D) 4–5 months old, (E) 2–3 months old, or (F) born during or just after the outbreak in January 2016 (month 0). Corrected optical density (COD) values are presented as percentage of a positive control serum.

In contrast to the data obtained on IgG, BRSV-specific IgA was only detectable in serum of a few animals and had mostly disappeared by 6–10 months after the outbreak (Fig 7). Strong responses were detected in single serum samples of two animals, 12 and 16 months after the outbreak in 2016 (id 606 and 620, Fig 7D and 7E). The analyses were repeated, and the IgA-peak persisted.

### BRSV-specific T cell responses in peripheral blood

No circulating BRSV-specific T cell responses were detected, either by lymphoproliferation assay or ELISPOT, in PBMC collected two months after infection and one year after infection.

### BRSV-specific IgA and IgG2 in nasal secretions

The local immunity to BRSV was studied in six individuals that were 4–5 months at the time of the first outbreak in 2016, from two months post the outbreak and for 4–24 months.

**Fig 8:**
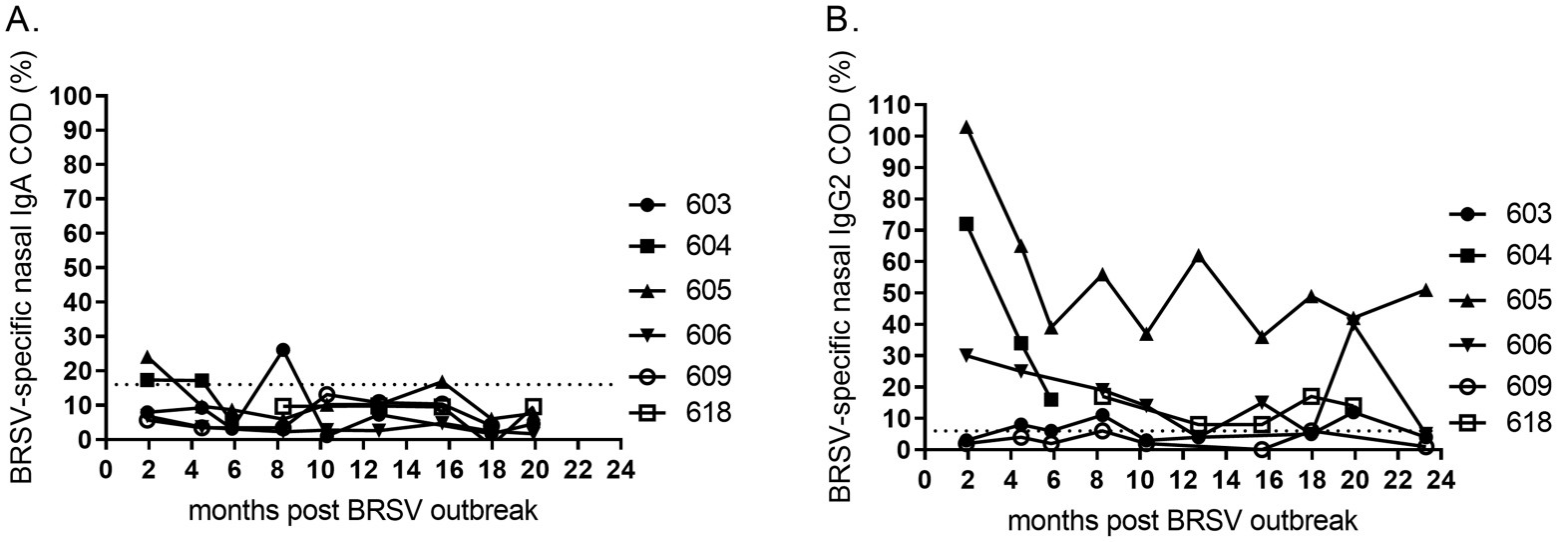
Kinetics of BRSV-specific antibodies in nasal secretions of calves following a BRSV outbreak. (A) Nasal BRSV-specific IgA and (B) nasal BRSV-specific IgG2 following BRSV-infection at 4–5 months of age in January 2016 (month 0). Corrected optical density (COD) values are presented as percentage of a positive control serum.

BRSV-specific IgA was detected in nasal secretions of all cattle two months post outbreak, but these responses were not stable over time (Fig 7). Whereas a drop was observed within six months, new peaks occurred later and were not coherent with the serum responses (Figs 7D and 8A). The BRSV-specific local IgG2 responses were more consistent. In agreement with the data on BRSV-specific IgG2 in serum, the highest levels of these antibodies were detected in the nasal secretions of calf no. 605 (Figs 4D and 8B). Similarly, IgG2 was detected in both serum and nasal secretions of calves no 604, 606 and 618.

### BRSV-specific IgG1 in milk

**Fig 9:**
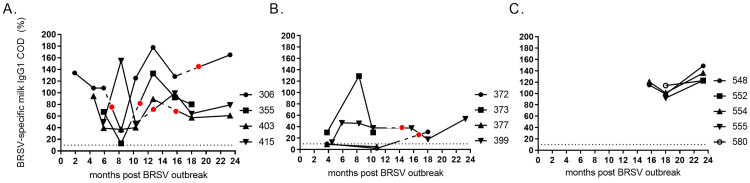
Kinetics of BRSV-specific total IgG1 in milk from cattle of different age and production status. At the time of a BRSV outbreak (in January 2016, month 0) the cattle were (A) 23–30 months old (<6 months gestation), (B) 24–26 months old (>6.5 months gestation), or (C) 7–11 months old. Corrected optical density (COD) values are presented as percentage of a positive control serum. Broken lines represent dry periods and red circles are timepoints for calving.

The levels of BRSV-specific IgG1 varied more in milk than in serum (Figs 3 and 9). Moreover, some cows that were in late gestation during the outbreak and had weak serum IgG responses, did not have detectable antibodies in milk on one or two occasions (id 372 and 377, Fig 9B).

### Reinfection and memory responses

The absence of BRSV re-circulation in the herd between the two outbreaks was confirmed by serology in 50 animals that were aged between 3 days and 22 months at sampling 26–27 months after the first outbreak. All animals that were between 3 months of age and 22 months of age were BRSV seronegative (n = 24, mean age 12 months, all born at least 5 months after the first outbreak), whereas 26/28 of the animals that were less than 3 months of age had BRSV-specific serum IgG1 (*i*.*e*. maternally derived).

**Fig 10:**
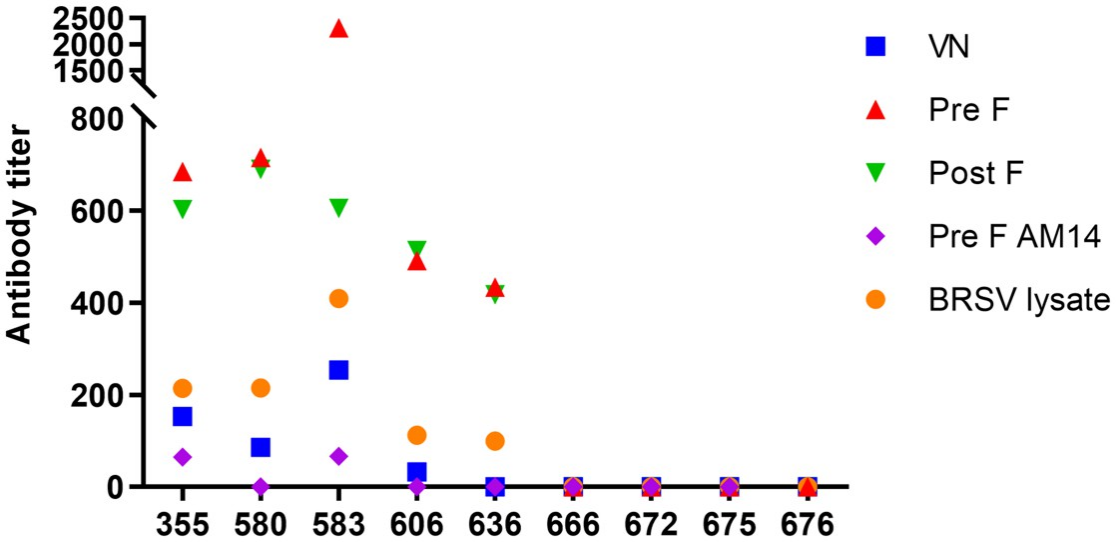
BRSV-specific serum antibodies in naïve cows and in cows infected with BRSV four years earlier. BRSV-neutralising antibodies (VN), BRSV-Pre F-specific IgG1 antibodies (Pre F) and BRSV-Post F-specific IgG1 antibodies (Post F), AM14-competing antibodies reactive to Pre F (AM14) and BRSV-specific IgG1 (BRSV lysate) 3 weeks before a BRSV (re)infection (March 2020). Animal 355–636 had been infected four years earlier (January 2016).

The serologic response of closely monitored animals, bled three weeks before (n = 9) and 2.5 months after (n = 8) the second outbreak in 2020, were further analysed in detail. Sera were screened by virus neutralisation test, by a commercial indirect IgG1-ELISA based on BRSV-infected cell-lysate and by indirect IgG1-ELISAs based on recombinant PreF- and PostF-protein. Moreover, a competitive ELISA based on recombinant PreF-protein and the human monoclonal antibody AM14, which recognizes a quaternary, prefusion trimer-specific epitope on the PreF [26], was used. Three weeks before the second outbreak, BRSV neutralising antibody titres and the BRSV-PreF- and -PostF-specific antibody titres were lower in cow 606 and 636, which were clearly not protected against reinfection three weeks later, compared to cow 355 and 580 that appeared partly protected (Fig 10, Table 3). Furthermore, animals in the youngest age group, which had served as sentinels for reinfections, were still BRSV-seronegative (Fig 10, animals 666–676). Virus detection was not attempted in this latter animal group because the animals were not accessible at the time of the second outbreak.

**Table 3:** Serum BRSV neutralising (VN) titre three weeks before detection of natural BRSV reinfection of cows, four years after a previous infection. BRSV RNA detected at reinfection in nasal secretions.

Cow id	Serum VN titre 3 weeks before reinfection	BRSV TCID_50_ eq. log10 (CT^a^) detected at reinfection
355	153	0.41 (38.3)
580	86	0.46 (38.1)
606	33	1.74 (33.2)
636	0	2.04 (32.1)

^a^ CT, cycle threshold value

**Fig 11:**
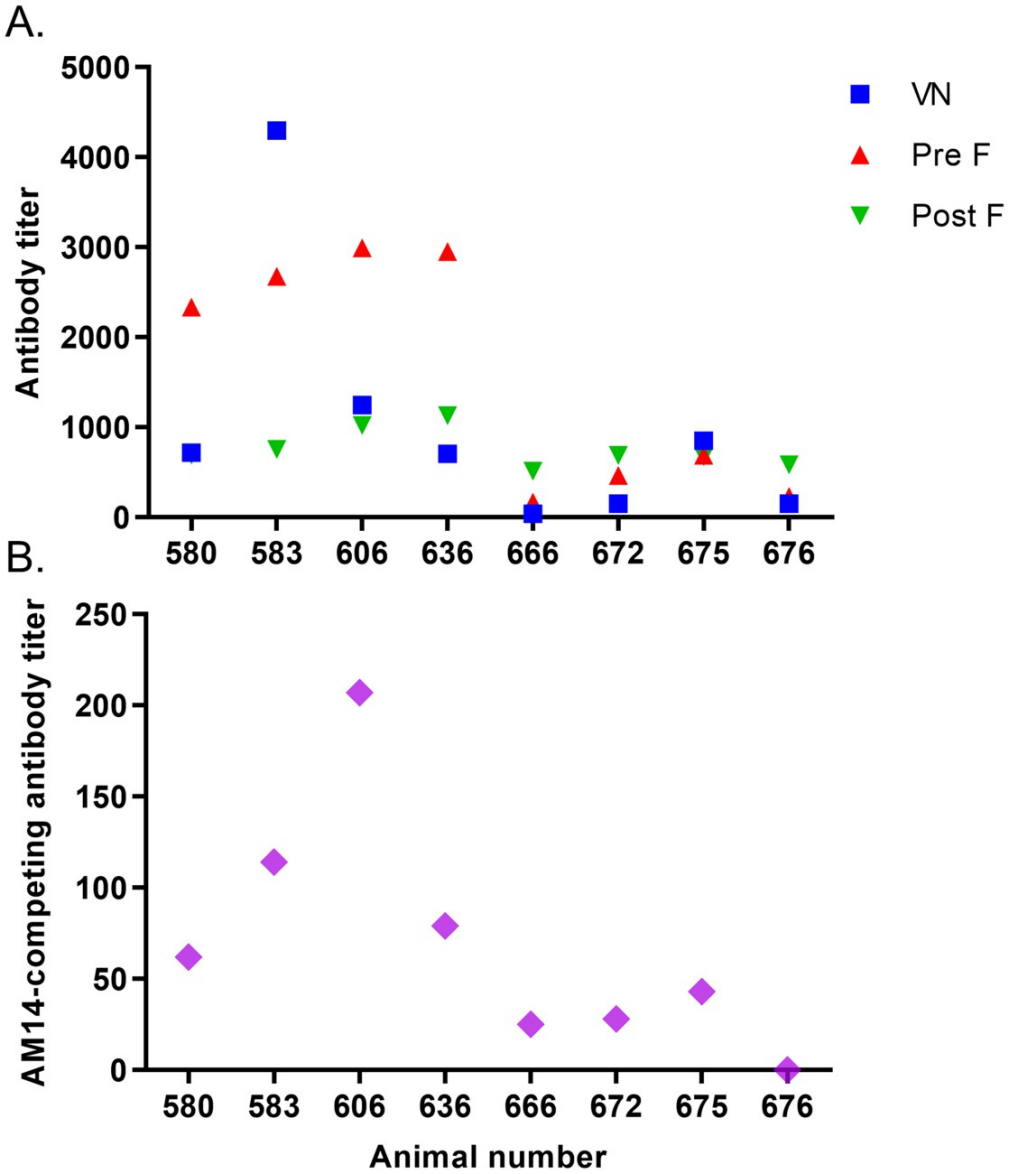
Primary antibody responses and memory responses to BRSV, 2.5 months after (re)infection in March 2020. (A) BRSV-neutralising antibodies (VN), BRSV-Pre F-specific IgG1 antibodies (Pre F), BRSV-Post F-specific IgG1 antibodies (Post F) and BRSV-specific IgG1 (BRSV lysate) in serum of cattle obtained in June 2020. (B) AM14 (reactive to a prefusion trimer-specific epitope on the PreF)-competing antibodies in the same animals and time point. Animal 580–636 had been infected four years earlier (January 2016) and animal 666–676 were previously naïve to infection.

Memory responses to BRSV, and in particular to the PreF protein, were detected in sera obtained 2.5 months after the second outbreak. The four animals that had experienced two BRSV infections (id 580, 583, 606 and 636) had significantly higher PreF- and PostF-specific responses, including AM14-competing antibodies, than the four animals that had experienced only one BRSV infection (id 666, 672, 675 and 676, p<0.0001, p<0.05 and p<0.05, respectively, Fig 11A and 11B) and had significantly stronger responses to the PreF form than to the PostF form of the BRSV F protein (p<0.01, Fig 11A). The mean neutralising responses were likewise higher in animals that had experienced two, compared to one infection (mean VN titre 1743 *vs*. 385), but this difference was not significant (p = 0.15, Fig 11A) due to a very strong response in one individual. Whereas the PreF-specific antibody titers increased 1.2–7 fold, the neutralising antibody titers increased 8–38 fold.

## Discussion

In this work the kinetics of BRSV-specific systemic and local immunity was monitored in a herd in which BRSV infections occurred only twice at a four-year interval. In contrast to BRSV-specific serum and nasal IgA and circulating T cell memory, serum IgG and BRSV-neutralising serum antibodies were long lasting. The humoral immune response directed against PreF, which previously was identified as key in protection against RSV [22, 27], was characterised by a strong memory response. A partial virological protection was obtained after four years, as indicated by reduced quantities of BRSV-RNA in nasal secretions.

The virus-specific immune response post infection is influenced by pre-existing immunity and the degree of infection, which at herd level influence the severity of an outbreak. The 2016 outbreak, which initiated the studied responses, was considered to be of moderate severity and representative of outbreaks observed on a regular basis. Less milk was sold because of decreased production, but also because of loss of cows and increased volumes of waste milk due to treatments. The reduction in milk production was highly significant and agrees with previous data from herds that presumably were recently infected with BRSV [28]. The simultaneous augmentation in milk fat indicates that cows were in negative energy balance, which may impede fertility, digestion and immunity [29, 30]. This can lead to disturbed stocking in different compartments of the herd, with subsequent health problems. The fertility could not be addressed because inseminations were performed by staff with different experience, but the immunity was investigated in detail.

Overall, BRSV-specific immunity was developed after the first outbreak because long-lasting BRSV-specific serum IgG antibody responses were observed in all studied animals that were born before the first outbreak. The duration of responses are in line with previous studies, in which virus neutralising antibodies as well as BRSV G and F-specific serum IgG1 and IgG2 antibodies lasted at least five to six months after natural primary BRSV infections [31, 32]. The present study confirms and extends these data since the studied period was longer and seronegative sentinel animals were regularly monitored to exclude reinfections.

It is very unlikely that virus circulated between the outbreaks, since no sentinel animal seroconverted and previously infected animals, which hypothetically would not have replicated the virus enough to produce a detectable humoral systemic response, would neither shed sufficient infectious virus to enable the transmission of virus. The antibody responses were very similar in animals of different ages, but two animal categories differed from the others: cows in late gestation and calves born during or after the outbreak. Cows in late gestation (the last 2 months before calving) developed weaker immune responses than other cows, maybe because of a physiologic immunosuppression that involves migration of antigen-presenting cells to the endometrium and an increase of γδT cells that have immuno-regulatory functions [33, 34]. Such cows should consequently not be targeted with vaccination. The calves that were born during or after the outbreak 2016 did not develop BRSV-specific IgG1, IgG2 or neutralising antibodies until 2020, either because they were not infected in 2016, or because of an inhibitory effect of BRSV-specific MDA. After the outbreak in 2020, these animals developed significantly lower antibody responses than older cows, which suggests that they had primary responses to BRSV and no previous infections.

The antibody levels were relatively stable over time in each animal. Nevertheless, some sporadic F- or N-reactive responses occurred in a few of the youngest animals between the two outbreaks. These reactions were not simultaneous or detected in the same individual, and therefore they were probably due to unspecific binding of serum proteins in the assays, or to cross-reactivity with antibodies directed to a protein in a closely related pathogen.

Virus neutralising serum antibodies play an essential role in protection and have been shown to prevent severe disease and occurrence of natural infections in humans [6, 7]. In all animals born before the outbreak, such antibodies increased by a wide (tenfold) range of titres after infection but dropped rapidly within 4 to 6 months and remained thereafter stable at moderate or low titres for at least four years. This initial drop in antibody titre, which was most striking in heifers, might have been due to a loss of short-lived plasmablasts, whereas the low but stable and durable antibody titres, observed in all age categories, indicate presence of long-lived plasma cells (LLPC) [35]. These latter cells are elicited in germinal centers and are not dependent on reactivation of memory cells [35].

The added effect of persistent antibody titres and memory responses was studied. Exposure to a previous infection significantly decreased virus shedding following reinfection after 50 months, although this needs to be verified by a larger dataset including kinetics of virus shedding. However, pre-existing neutralising titres up to at least 1:153 did not afford complete virological protection. One individual with titres of 1:254 was likewise re-infected, since strong memory responses occurred, but virus shedding was not investigated.

The neutralising antibody titres that were non-protective against infection were reached in many cases within six months to one year. Although other immunological parameters, such as tissue resident memory lymphocytes, probably play an important role at this stage [36, 37], it seems that there is a direct or indirect association between serum virus neutralising antibodies and virus re-excretion after natural BRSV reinfection.

Subunit vaccines based on PreF are potent inducers of protective immune responses, including neutralising antibodies [38, 39]. While PreF- and PostF-specific serum antibody titres did not differ significantly four years after infection, the memory to PreF was significantly stronger than that to PostF, following re-infection. This might be due to affinity maturation among memory B cells, which probably did not contribute to the pool of LLPC that produced antibodies before reinfection, and underlines the efficacy of PreF-specific antibodies compared to PostF-specific antibodies. Overall, these data confirm that PreF is a key target to get a long lasting immunity and memory.

In agreement with data in vaccinated calves and in naturally infected children [11, 27], virus-specific cellular responses were not detectable in the peripheral circulation of our cattle 2 months post infection. Similarly, serum IgA responses were only detectable in some animals and were mostly of short duration. Both IgA and T cell responses in peripheral blood were previously used as criteria to evaluate vaccine efficacy in calves [22], however, taking these new observations into account, neutralising responses seem to be a more important parameter in the development of vaccines with long duration of protection. For economic reasons, the duration of protection is an essential criterion for cattle vaccines.

Local (respiratory) anamnestic BRSV-specific IgA might additionally be important, especially at the peak of immunity [17, 19], but the IgA memory seems to be short, at least when induced by live intranasal vaccines [27]. Unfortunately, nasal secretions for antibody analyis were not collected after reinfection in this study. The sparse IgA peaks observed, both in serum and nasal secretions, might be explained by the existence of poly-reactive natural antibodies against conserved epitopes on microbes [40], because the cell clones that produces such antibodies are enriched among IgA-secreting plasma cells [41]. Another hypothesis is that BRSV-specific IgA responses were triggered by persistent antigen in BRSV carrier animals, as has been shown for foot and mouth virus [42]. Nevertheless, despite that persistent BRSV has previously been detected in lymphoid tissue [43], re-excretion of persistent virus from carrier cattle has not been demonstrated [44, 45], neither for BRSV, nor for FMDV, and there is no indication that virus spread from any potential carrier animal in this herd.

Variation over time was also observed for BRSV-specific IgG1 in milk. This inconsistency, which might be explained by difference in milk yield and the resulting varying degree of antibody dilution over time, impedes the possibility to screen milk for protective immunity to identify targets for vaccination.

Since experimental vaccines based on only the PreF protein have demonstrated good efficacy in calves [22, 27, 38], the N protein could potentially serve as DIVA protein in the future. The detection of N-specific antibodies in PreF-vaccinated animals could thereby be used to identify the occurrence of BRSV infections and indicate when the duration of vaccine-induced protective immunity has waned. The antibody responses to the N protein were in general strong and long-lasting, which indicate a potential use of this assay as DIVA-test.

In conclusion, this study demonstrated a significant impact of BRSV infections on food intake and milk production in dairy cows. The infections induced long-lasting systemic humoral responses and a strong immunological memory to the PreF protein. Despite this immunity, adult cattle became re-infected and this resulted in mild respiratory clinical signs and virus shedding. The role of re-infected adults in virus circulation should therefore be investigated further. If confirmed, based on the demonstrated PreF-memory and if shown to be cost effective, regular PreF-vaccination might aid to rupture the circulation of this virus.

## Supporting information

S1 Fig BRSV RNA detected in nasal swabs of cows that were naturally re-infected four years after a previous natural BRSV infection (n = 7, reinfection) and cows that were naturally infected for the first time (n = 5). Samples were collected once and on the same day. All cows were housed in the same section of the loose range system. The unit TCID_50_ equivalent (TCID_50_ eq.) was used since the standard curve used in the assay was based on a BRSV-infected cell lysate with a known titre. Error bars represent standard deviation.
(TIF)

S1 Table Least Square Means of milk production, roughage and non-roughage intake of 272, 163 and 272 cows, respectively, that remained in production for commercialisation, before, during and after a BRSV outbreak in 2016. (DOCX)

S2 Table Differences of Least Squares Means of milk production, roughage and non-roughage intake of 272, 163 and 272 cows, respectively that remained in production for commercialisation, before, during and after a BRSV outbreak. (DOCX)
