# Supplementary figures and images for: Longitudinal study of the immune response and memory following natural bovine respiratory syncytial virus infections in cattle of different age
Source: PLoS One. 2022 Sep 16;17(9):e0274332. doi: 10.1371/journal.pone.0274332 (PMC9481050; doi:10.1371/journal.pone.0274332)

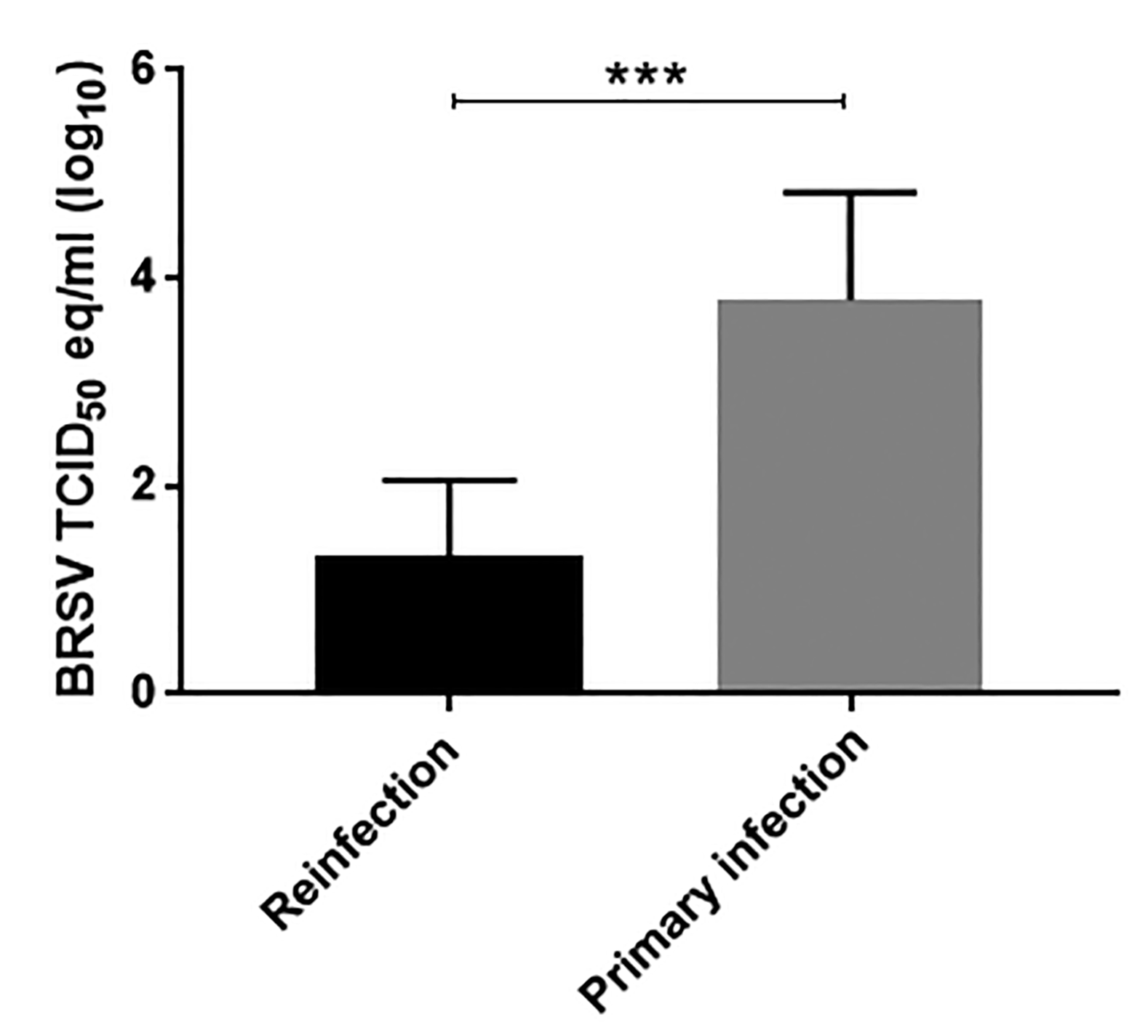

Supplement: S1 Fig — Samples were collected once and on the same day. All cows were housed in the same section of the loose range system. The unit TCID50 equivalent (TCID50 eq.) was used since the standard curve used in the assay was based on a BRSV-infected cell lysate with a known titre. Error bars represent standard deviation. (TIF) [file pone.0274332.s001.tif]
