# Supplementary material for: Longitudinal study of the immune response and memory following natural bovine respiratory syncytial virus infections in cattle of different age
Source: PLoS One. 2022 Sep 16;17(9):e0274332. doi: 10.1371/journal.pone.0274332 (PMC9481050; doi:10.1371/journal.pone.0274332)
Supplement: S1 Table — (DOCX) [file pone.0274332.s002.docx]

*Supplement 2. Least Square Means of milk production, roughage and non-roughage intake of 272, 163 and 272 cows, respectively, that remained in production for commercialisation, before, during and after a BRSV outbreak in 2016.*

| *Parameter* | *Phase^a^* | *Mean value (kg)* | *SE* |
| --- | --- | --- | --- |
| *Milk production* | Pre | 30.1 | 0.636 |
|  | Base | 29.7 | 0.639 |
|  | Inter | 28.9 | 0.639 |
|  | **Outbreak** | **27.0** | 0.634 |
|  | Post | 28.7 | 0.632 |
| *Roughage intake* | Pre | 36.2 | 0.879 |
|  | Base | 36.1 | 0.865 |
|  | Inter | 37.4 | 0.889 |
|  | **Outbreak** | **33.4** | 0.844 |
|  | Post | 38.9 | 0.871 |
| *Non-roughage intake* | Pre | 8.9 | 0.270 |
|  | Base | 9.0 | 0.274 |
|  | Inter | 8.4 | 0.272 |
|  | **Outbreak** | **8.3** | 0.270 |
|  | Post | 9.1 | 0.268 |

^a^ Time periods were defined as follows: Pre (December 1, 2015 to December 14, 2015); Base (December 15, 2015 to December 22, 2015); Inter (December 23, 2015 to January 2, 2016); Outbreak (January 3, 2016 to January 15, 2016); Post (January 16, 2016 to January 31, 2016)

SE, Standard error
