# Supplementary material for: Longitudinal study of the immune response and memory following natural bovine respiratory syncytial virus infections in cattle of different age
Source: PLoS One. 2022 Sep 16;17(9):e0274332. doi: 10.1371/journal.pone.0274332 (PMC9481050; doi:10.1371/journal.pone.0274332)
Supplement: S2 Table — (DOCX) [file pone.0274332.s003.docx]

*Supplement 3. Differences of Least Squares Means of milk production, roughage and non-roughage intake of 272, 163 and 272 cows, respectively that remained in production for commercialisation, before, during and after a BRSV outbreak*

| *Parameter* | *Phase* | *Phase* | *Estimate* | *SE* | *DF* | *t Value* | *Pr > t* | *Adj P* |
| --- | --- | --- | --- | --- | --- | --- | --- | --- |
| Milk production | Base | Inter | 0.8165 | 0.365 | 836 | 2.24 | 0.0253 | 0.1660 |
|  | Base | Post | 1.0326 | 0.367 | 846 | 2.81 | 0.0050 | 0.0403 |
|  | Base | Pre | -0.3836 | 0.364 | 841 | -1.05 | 0.2924 | 0.8301 |
|  | **Base** | **Outbreak** | 2.7436 | 0.364 | 840 | 7.53 | <.0001 | **<.0001** |
|  | Inter | Post | 0.2160 | 0.365 | 844 | 0.59 | 0.5539 | 0.9763 |
|  | Inter | Pre | -1.2001 | 0.368 | 843 | -3.26 | 0.0012 | 0.0102 |
|  | **Inter** | **Outbreak** | 1.9270 | 0.362 | 838 | 5.33 | <.0001 | **<.0001** |
|  | **Post** | **Pre** | -1.4161 | 0.371 | 854 | -3.82 | 0.0001 | **0.0014** |
|  | **Post** | **Outbreak** | 1.7110 | 0.353 | 840 | 4.85 | <.0001 | **<.0001** |
|  | **Pre** | **Outbreak** | 3.1271 | 0.368 | 848 | 8.50 | <.0001 | **<.0001** |
| Roughage consumption | Base | Inter | -1.2720 | 0.841 | 459 | -1.51 | 0.1309 | 0.5543 |
|  | Base | Post | -2.7606 | 0.870 | 489 | -3.17 | 0.0016 | 0.0138 |
|  | Base | Pre | -0.09307 | 0.814 | 448 | -0.11 | 0.9090 | 1.0000 |
|  | **Base** | **Outbreak** | 2.7264 | 0.819 | 471 | 3.33 | 0.0009 | **0.0083** |
|  | Inter | Post | -1.4886 | 0.870 | 474 | -1.71 | 0.0876 | 0.4277 |
|  | Inter | Pre | 1.1789 | 0.858 | 462 | 1.37 | 0.1700 | 0.6444 |
|  | **Inter** | **Outbreak** | 3.9984 | 0.821 | 457 | 4.87 | <.0001 | **<.0001** |
|  | Post | Pre | 2.6675 | 0.888 | 491 | 3.00 | 0.0028 | 0.0234 |
|  | **Post** | **Outbreak** | 5.4870 | 0.810 | 460 | 6.77 | <.0001 | **<.0001** |
|  | **Pre** | **Outbreak** | 2.8195 | 0.837 | 474 | 3.37 | 0.0008 | **0.0073** |
| Non-roughage consumption | Base | Inter | 0.5587 | 0.198 | 844 | 2.82 | 0.0050 | 0.0398 |
|  | Base | Post | -0.07250 | 0.199 | 855 | -0.36 | 0.7155 | 0.9962 |
|  | Base | Pre | 0.1172 | 0.200 | 853 | 0.59 | 0.5569 | 0.9769 |
|  | **Base** | **Outbreak** | 0.7145 | 0.199 | 851 | 3.59 | 0.0003 | **0.0032** |
|  | Inter | Post | -0.6312 | 0.197 | 854 | -3.21 | 0.0014 | 0.0120 |
|  | Inter | Pre | -0.4415 | 0.199 | 855 | -2.22 | 0.0266 | 0.1729 |
|  | Inter | Outbreak | 0.1558 | 0.197 | 849 | 0.79 | 0.4280 | 0.9327 |
|  | Post | Pre | 0.1897 | 0.199 | 866 | 0.96 | 0.3395 | 0.8748 |
|  | **Post** | **Outbreak** | 0.7870 | 0.191 | 848 | 4.12 | <.0001 | **0.0004** |
|  | Pre | Outbreak | 0.5973 | 0.1990 | 862 | 3.00 | 0.0028 | 0.0231 |

Estimate, difference in mean value between phases; SE, Standard error; DF, degrees of freedom; t Value, Estimate/SE; Pr > t, significance for the effect of phase on estimate; Adj P, Adjusted P value (Tukey-Kramers test)
